# Natural Vegetation Phenology in Central Asia: Satellite-Derived Trends and Nonlinear Dynamics via EEMD

**DOI:** 10.3390/biology15141175

**Published:** 2026-07-17

**Authors:** Gang Long, Anming Bao, Tao Yu, Tao Li, Fengjiao Song, Sulei Naibi, Yalong Li, Ye Yuan, Xiaoran Huang

**Affiliations:** 1 National Key Laboratory of Ecological Security and Sustainable Development in Arid Region, Xinjiang Institute of Ecology and Geography, Chinese Academy of Sciences, Urumqi 830011, China; 2 University of Chinese Academy of Sciences, Beijing 100049, China; 3 Key Laboratory of GIS & RS Application Xinjiang Uygur Autonomous Region, Urumqi 830011, China

**Keywords:** vegetation phenology, SOP, satellite-derived NDVI, climate change, arid and semi-arid ecosystems, EEMD

## Abstract

Understanding vegetation phenology is critical for assessing the impacts of climate change, particularly in regions vulnerable to environmental fluctuations. This study investigates the temporal trends and spatial variability of the start of photosynthetic activity (SOP) in natural vegetation across Central Asia over the past four decades (1982–2022) using satellite-derived Normalized Difference Vegetation Index (NDVI) data. The research emphasizes the critical role of vegetation phenology in understanding responses to climate change. To extract spring phenological data, various smoothing techniques were applied, including filter-based methods, asymmetrical Gaussian fitting, and three nonlinear and piecewise linear methods, ensuring accurate representation from continuous time series data. NDVI time series were smoothed using a phenological extraction package. The results indicate an average advance in SOP of 1.26 days per decade, with forest ecosystems exhibiting the greatest shift at 3.05 days per decade. A spring temperature threshold near 0 °C was identified as a reliable predictor for dormancy break. Ensemble Empirical Mode Decomposition (EEMD) was utilized to differentiate between cumulative and instantaneous trends, revealing dynamic phenological responses. A notable shift around 2005 was observed, with approximately 76.28% of pixels showing a change in SOP trend, while 23.60% displayed a stable, monotonic trend. Vegetation at elevations between 1500 and 3000 m experienced a significant SOP advance of 2.27 days per decade, whereas vegetation above 3000 m showed no significant change over the study period. Spatially, SOP trends exhibited a latitudinal gradient, with delays observed in the southern regions and advancements north of 45° N. These findings underscore the importance of developing region-specific phenological models to inform environmental management and climate adaptation strategies, particularly in arid and semi-arid ecosystems.

## Simple Summary

Spring green-up marks the beginning of seasonal vegetation activity and influences ecosystem carbon uptake, water use, and productivity. However, long-term phenological changes in natural vegetation across arid Central Asia remain uncertain because previous studies have generally emphasized linear trends and have rarely examined whether trends reverse over time or differ among vegetation types, elevations, and climatic zones. We analyzed satellite vegetation observations and meteorological records from 1982 to 2022 to determine when natural vegetation began photosynthetic activity and how this timing changed. Spring activity generally began earlier, but the direction and magnitude of change differed among forests, grasslands, shrublands, and sparse vegetation. A widespread change in phenological trends was also identified around 2005. Winter cold accumulation, spring warming, precipitation, latitude, and elevation jointly contributed to the regional differences. These findings indicate that a single uniform phenological model is unsuitable for Central Asia and that vegetation- and region-specific models are required for ecological forecasting and climate-adaptation planning.

## 1. Introduction

Vegetation phenology is a vital indicator of seasonal and interannual variations in environmental conditions, reflecting the dynamic responses of terrestrial ecosystems to climate change [1,2,3]. Phenological shifts, such as changes in the start of photosynthetic activity (SOP), are particularly important as they capture the impact of global climate change on ecosystem function and structure [4,5,6]. Satellite remote sensing, which measures canopy reflectance across large spatial and temporal scales, has become an indispensable tool for monitoring vegetation phenology [7,8]. Over the past century, and especially in the last two decades, global mean surface temperature has increased significantly, making climate warming a critical environmental issue [9]. The timing of vegetation’s SOP, which is strongly influenced by meteorological and climatic factors, is a key parameter for studying the effects of global changes on terrestrial ecosystems and their feedback mechanisms [10].

Central Asia, situated in the middle and high latitudes of the Northern Hemisphere, is a region particularly sensitive to climate change [11,12,13]. Studying the changes in SOP and its relationship with surface temperature in this area is crucial for understanding how ecosystems respond to changing climatic conditions. Remote sensing-based land surface phenology (LSP), which extracts phenological metrics from satellite observations at the pixel scale, offers valuable insights into the temporal dynamics of vegetation phenology across large regions [14,15,16]. However, previous studies have primarily focused on broad vegetation functional types, often overlooking the nuanced phenological responses of native vegetation and the impacts of land use and land cover change (LULCC) on these responses [17,18]. For example, ref. [17] compared three satellite-derived measures of the start of the growing season and matched them to field data from the Harvard Forest in Massachusetts, revealing that each method provides a reasonably accurate representation of surface phenology. Similarly, ref. [18] used MODIS-derived vegetation products to compare field measurements of forest canopy phenology in northern Wisconsin, highlighting the utility of remote sensing in capturing springtime phenological events.

Land use refers primarily to the ways in which humans utilize and manage land, whereas land-cover change may result from both anthropogenic activities and natural ecosystem transitions, including climate-driven shifts in vegetation composition [19,20,21]. The present study does not directly quantify historical land-use or land-cover change. Instead, a land-cover product is used to classify natural vegetation into forest, grassland, shrubland, and sparse-vegetation categories for stratified phenological analysis [22,23].

Accordingly, this study aims to: (1) quantify the spatial distribution and 1982–2022 trends of SOP in natural vegetation across Central Asia; (2) compare phenological changes among vegetation types, aridity classes, elevation bands, and latitudinal zones; (3) distinguish monotonic changes and temporal turning points using EEMD; and (4) evaluate the associations of SOP with winter chilling, spring thermal forcing, and preseason precipitation. We hypothesize that temperature-limited forest and grassland ecosystems exhibit stronger SOP advancement than moisture-limited shrubland and sparse vegetation and that complex topography weakens simple latitudinal patterns.

## 2. Materials and Methods

The analytical framework consisted of six stages. First, land-cover, vegetation-index, meteorological, elevation, and aridity datasets were harmonized to a common spatial grid. Second, non-target land-cover classes were masked and invalid or anomalously low NDVI observations were identified and corrected. Third, annual NDVI curves were reconstructed and SOP was extracted using the verified threshold rule. Fourth, winter chilling, spring thermal forcing, and preseason precipitation were calculated for each pixel and year. Fifth, conventional linear and EEMD-based trend metrics were estimated. Finally, phenological patterns and climatic associations were summarized by vegetation type, aridity class, elevation, latitude, and administrative region.

### 2.1. Study Area

**Figure 1:**
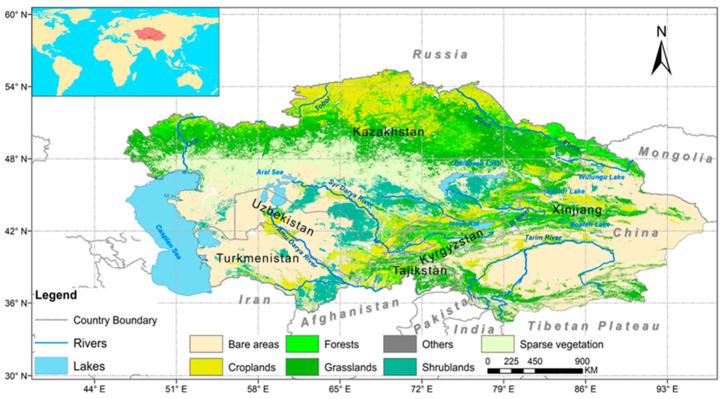
Location, administrative boundaries, and land-cover distribution of the study area. The study domain includes Kazakhstan, Uzbekistan, Kyrgyzstan, Tajikistan, Turkmenistan, and the Xinjiang Uyghur Autonomous Region of China. For the natural-vegetation classification, dry-steppe pixels were incorporated into the grassland category; therefore, the grassland class displayed in the map includes dry-steppe vegetation.

The study domain follows the administrative boundaries of Kazakhstan, Uzbekistan, Kyrgyzstan, Tajikistan, Turkmenistan, and the Xinjiang Uyghur Autonomous Region of China. The coordinate range of 34.3–55.4° N and 46.5–96.4° E represents only the outer geographical extent of the study area; locations within this rectangular extent but outside the specified administrative boundaries were excluded (Figure 1). This region contains vast expanses of temperate deserts, shaped by its unique inland geographical location and topographical features. The complex and diverse landforms, coupled with significant regional climatic variability and uneven water resource distribution, contribute to the fragility of its ecosystems.

Based on the Climate Change Initiative–Land Cover (CCI-LC) product released by the European Space Agency in 2020, bare land constitutes 35.26% of the total study area, primarily concentrated in the southwestern and southeastern regions. Dry steppe, the second most prevalent land cover type, accounts for 24.00% of the area. Sparse vegetation covers 13.44%, while dry cropland occupies 6.15%, predominantly in northern Kazakhstan. Irrigated cropland, representing 5.97%, is mainly found in the southern part of the region. Shrubland constitutes 5.28% of the area, whereas wetlands and forests are less common, covering only 0.29% and 1.49%, respectively. Water bodies and permanent ice and snow account for 2.85%, and urban areas make up 0.19%. The distribution of natural vegetation types across the five Central Asian countries generally follows a latitudinal gradient, while vegetation in the Tianshan-Pamir-Kunlun mountain ranges is more influenced by elevation.

For the subsequent phenological analysis, the original land-cover classes were reclassified into four natural-vegetation categories: forest, grassland, shrubland, and sparse vegetation. Dry steppe was incorporated into the grassland category; therefore, the grassland class shown in Figure 1 and used throughout the manuscript includes dry-steppe vegetation.

### 2.2. Data Sources

Land-cover information was obtained from the 2020 European Space Agency Climate Change Initiative Land Cover product. Since our study focused on Central Asia, we chose 4 types of natural vegetation (forest, grassland, shrub and sparse vegetation) as shown in Figure 1. These data are available at http://esa-landcover-cci.org/?q=node/175 (accessed on 25 October 2024).

Satellite-derived SOP dates were obtained from the Advanced Very High-Resolution Radiometer (AVHRR), Normalized Difference Vegetation Index (NDVI) dataset developed by the Global Inventory Modeling and Mapping Studies (GIMMS) group. This dataset is widely used in vegetation studies due to its long-term coverage and high quality. The monthly mean NDVI data (GIMMS-NDVI3g), processed using Maximum Value Composites (MVC), has an original spatial resolution of 8 km and a temporal resolution of 15 days. The data are publicly accessible at https://doi.org/10.3334/ORNLDAAC/2187 http://esa-landcover-cci.org/?q=node/175 (accessed on 25 October 2024).

Hourly 2-m air temperature and total precipitation data for 1982–2022 were obtained from the ERA5 hourly data on single levels, produced by the European Centre for Medium-Range Weather Forecasts (ECMWF) and distributed through the Copernicus Climate Change Service Climate Data Store. The dataset has an hourly temporal resolution and a spatial resolution of 0.25° × 0.25°. Local sunrise and sunset times were calculated for each grid cell based on its longitude and latitude, and the hourly 2-m air temperature records within the daylight period were used to derive daytime minimum and maximum temperatures. Hourly total precipitation was summed to obtain daily precipitation totals, which were subsequently summarized over the defined preseason period. The temperature and precipitation data were spatially aligned with the GIMMS NDVI grid before the pixel-level analyses.

### 2.3. Extraction of Phenology

Quality-control flags supplied with the GIMMS dataset were first used to exclude fill values and unreliable observations according to the product documentation [24]. For pixel ppp and year yyy, the annual minimum positive valid NDVI was calculated as:

mp,y=tmin{NDVIp,y,t:NDVIp,y,t>0 and the observation is valid}.

The pixel-specific background NDVI was subsequently calculated as:

Bp=mediany (mp,y).

Each observation was corrected according to:

$$NDVI_{p,y,t}^{*}=\left\{\begin{array}{cc} B_{p}, & NDVI_{p,y,t}<B_{p}\\ NDVI_{p,y,t}, & NDVI_{p,y,t}>B_{p} \end{array}\right.$$

This procedure reduced the influence of anomalously low observations caused by snow, cloud contamination, atmospheric effects, and residual sensor noise. If no independent snow-cover mask was used, the correction should be interpreted as reducing, rather than completely eliminating, snow-related contamination.

To extract spring phenological data, a variety of smoothing techniques were applied to the time series data [25,26], including filter-based smoothing (e.g., Savitzky–Golay filter), HANTS asymmetrical Gaussian fitting, nonlinear fitting methods, and piecewise linear fitting. The NDVI time series was refactored using the phenological extraction package available at https://github.com/antonkout/Phenology-Extraction http://esa-landcover-cci.org/?q=node/175 (accessed on 25 October 2024). Inflection point detection methods identified local maxima or minima in the smoothed NDVI curve, while threshold methods were used to compare the NDVI series against fixed or dynamic thresholds. The fixed threshold (20% of the annual maximum NDVI) was found to be particularly suitable for extracting spring phenology in the arid regions of Central Asia.

Pixels classified as bare land, cropland, water, urban land, or permanent snow and ice were excluded because the study focused on natural vegetation. Pixels with insufficient valid NDVI observations, low seasonal amplitude, or unsuccessful curve fitting were also assigned NoData.

### 2.4. Determination of Dormancy Release and Spring Thermal Accumulation

Winter cold accumulation and subsequent spring thermal accumulation represent two consecutive stages of vegetation phenological development. Winter cold exposure contributes to the fulfilment of chilling requirements and the release of vegetation from dormancy, whereas growing degree days (GDDs) represent the heat accumulated after dormancy release and before the onset of photosynthetic activity [27,28].

For each pixel and year, the daily temperature series from the preceding autumn through spring was examined to identify the end of winter dormancy. Dormancy release was defined as the date on which the daily mean temperature first reached the empirically determined temperature threshold closest to 0 °C. This threshold was selected because it showed the closest relationship with the remotely sensed onset of spring vegetation activity across the study area [29,30,31].

The date of dormancy release was also used as the starting date for GDD accumulation. Thus, GDDs were accumulated from the first day on which the daily mean temperature reached the identified dormancy-release threshold until the SOP date derived from the NDVI time series:

$$GDDy=\sum_{d=d0,y}^{SOPy}max\left[Tmean,y\left(d\right)-Tb,0\right],$$

where GDDy is the accumulated thermal forcing in year y, d_0_, y is the date of dormancy release, SOPy is the remotely sensed SOP date, T_mean,y_(d) is the daily mean air temperature on day ddd, and TbT_bTb is the identified base-temperature threshold near 0 °C. Temperatures below T_b_ contributed zero to the accumulated GDD.

Accordingly, winter cold accumulation was evaluated for the period preceding dormancy release, whereas GDD represented the thermal forcing accumulated after dormancy release. This separation avoids treating winter chilling and spring heat accumulation as the same process and clarifies how GDD was used to characterize the temperature requirement for vegetation green-up.

### 2.5. Linear and EEMD-Based Trend Analysis

The conventional interannual trend in the SOP was first quantified for each valid pixel using ordinary least-squares linear regression, with calendar year as the independent variable and annual SOP, expressed as day of year, as the dependent variable:

SOPt=α+βt+εt

where SOP_t_ represents the SOP in year ttt, α\alphaα is the regression intercept, β\betaβ is the temporal slope, and εt\varepsilon_tεt is the residual error. The slope β\betaβ, initially expressed in days per year, was multiplied by 10 and reported in days per decade. Because an earlier SOP corresponds to a smaller day-of-year value, a negative slope indicates SOP advancement, whereas a positive slope indicates SOP delay. The statistical significance of the temporal trend was evaluated using a two-sided Student’s *t*-test under the null hypothesis that the regression slope was equal to zero. Trends were considered statistically significant at *p* < 0.05. The coefficient of determination (R2R^) was used to evaluate the goodness of fit of the linear regression.

Although linear regression provides an overall rate of change for the complete study period, it cannot adequately represent nonlinear variation or changes in trend direction. Therefore, Ensemble Empirical Mode Decomposition (EEMD) was further applied to the annual SOP series. EEMD is a noise-assisted extension of Empirical Mode Decomposition that separates a nonlinear and non-stationary time series into several oscillatory intrinsic mode functions and a residual long-term component:

$$SOP(t)=\sum_{k=0}^{k}IMFk(t)+R(t)$$

where IMFk(t) is the k intrinsic mode function, K is the total number of decomposed components, and R(t) is the residual component representing the long-term nonlinear trend. By adding white noise to the original series, repeatedly decomposing the noise-assisted series, and averaging the resulting components, EEMD reduces mode mixing and improves the stability of the extracted long-term trend.

The cumulative SOP change was calculated relative to the beginning of the study period:

C(t)=R(t)−R(1982)

where C(t) represents the cumulative SOP change in year t. A negative cumulative value indicates that SOP occurred earlier than in 1982, whereas a positive value indicates that SOP occurred later.

The instantaneous rate of change was calculated from the first derivative of the EEMD residual trend:

$$I(t)=\frac{dR(t)}{dt}$$

where I(t) represents the rate and direction of SOP change at a specific time. Negative instantaneous values indicate a temporal tendency toward earlier SOP, whereas positive values indicate a tendency toward later SOP. In contrast to the full-period linear slope, the instantaneous rate allows the direction and magnitude of phenological change to vary through time.

Turning points were identified when the instantaneous rate changed sign and the EEMD residual trend exhibited a corresponding local maximum or minimum. Based on the temporal behavior of the residual trend, each valid pixel was classified into one of four categories: monotonic advance, monotonic delay, delay followed by advance, or advance followed by delay. A transition from positive to negative instantaneous values represents a shift from SOP delay to advancement, whereas a transition from negative to positive values represents a shift from advancement to delay.

**Figure 2:**
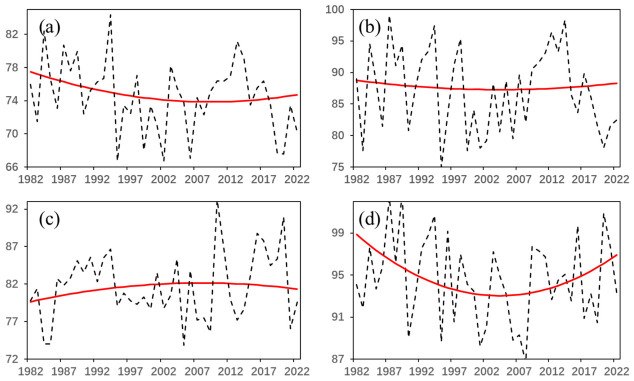
Example of EEMD’s decomposition of SOP time series into oscillatory components and long-term trends. (**a**) Represents SOP cumulative trend with SG filter. (**b**) Represents the SOP cumulative trend with Gauss filter. (**c**) Represents SOP cumulative trend with HANTS filter. (**d**) Represents SOP cumulative trend with DL filter.

The linear and EEMD-based analyses provide complementary information. An example of EEMD’s decomposition of SOP time series into oscillatory components and long-term trends is shown in the Figure 2. Linear regression quantifies the average rate of SOP change over 1982–2022, whereas EEMD identifies nonlinear temporal evolution, trend reversals, and turning points that may be obscured by a single full-period slope.

#### 2.5.1. Cumulative Trend and Trend Classification

The cumulative trend represents the overall change between a specific time point, *t*, and a reference time, *t*_0_, as defined by the EEMD method. According to Wu and Huang (2009) [32], the cumulative trend is either monotonic or exhibits only one extreme point (turning point). Therefore, the cumulative trend of the SOP can be categorized into four types: monotonic delay, monotonic advance, transition from delay to advance, and transition from advance to delay. Similarly, the cumulative trend of preseason cold accumulation can be classified into four patterns: monotonic upward trend, monotonic downward trend, transition from upward to downward trend, and transition from downward to upward trend.

#### 2.5.2. Instantaneous Rate of Change

The instantaneous trend reflects the rate of change at any given time *t*, offering insights into the real-time dynamics of long-term trends. This method provides a more nuanced understanding of temporal changes compared to traditional linear trend analysis. By applying the instantaneous trend, we were able to explore the relationship between SOPs and preseason cold accumulation, revealing real-time interactions that would be difficult to capture using linear assumptions.

## 3. Results

### 3.1. Phenological Distribution Characteristics

The vegetation growing season in Central Asia exhibits two distinct patterns in the spatial distribution of the SOP. First, from northern Kazakhstan to southern Turkmenistan, there is a clear trend of progressively earlier SOP from north to south. This pattern corresponds with the zonal distribution of land surface temperature, where warmer temperatures at lower latitudes drive earlier vegetation green-up. The latitudinal temperature gradient is the primary driver of this trend.

**Figure 3:**
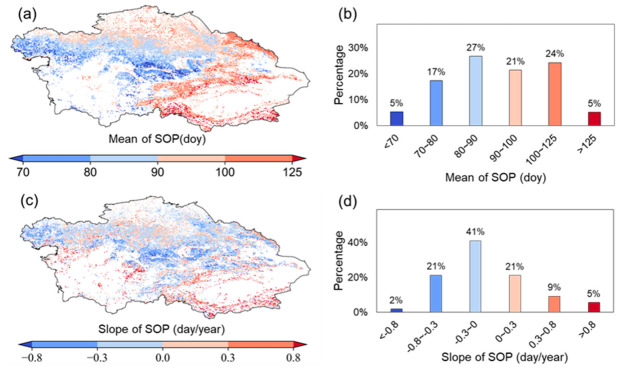
Spatial distribution and frequency of natural-vegetation SOP. (**a**) Mean SOP during 1982–2022; (**b**) frequency distribution of mean SOP; (**c**) linear SOP trend; and (**d**) frequency distribution of SOP trends. White areas represent non-target land-cover classes that were intentionally masked, including bare land, cropland, water, urban land, and permanent snow or ice, as well as pixels with insufficient valid observations or failed SOP retrieval.

Second, in the regions of Kyrgyzstan, Tajikistan, and most of Xinjiang, China, the latitudinal influence on SOP is much weaker. In these areas, terrain and other local environmental factors, such as elevation and topographic variability, exert a stronger influence on the timing of SOP. The complex topography in these mountainous regions creates microclimates that modulate phenological responses, thereby diminishing the typical north–south zonality observed in other parts of Central Asia. The annual SOP series for the four vegetation classes is shown in Figure 3.

**Figure 4:**
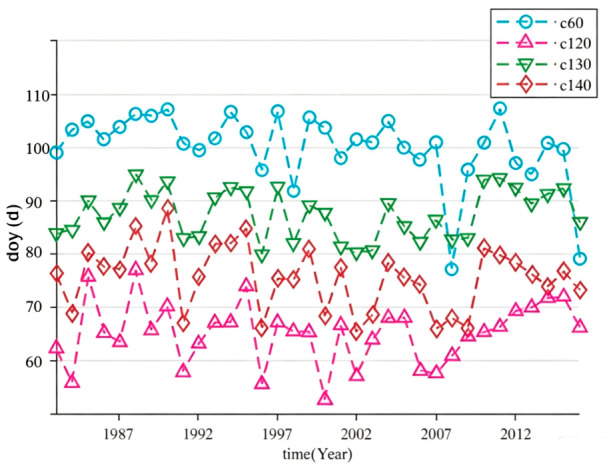
Variation trend of SOP of different cover types in the study area during 1982–2022.

From an inter-annual perspective, the average SOP in the study area advanced at a rate of 1.26 days per decade (Figure 4).

Among different vegetation types, forest ecosystems exhibited the most pronounced advancement, with SOP shifting 3.05 days earlier per decade. Grassland vegetation showed a similar trend, advancing at 1.44 days per decade. In contrast, shrub and sparse vegetation types exhibited delayed SOP trends, with shifts of 0.26 days per decade and 0.35 days per decade, respectively.

**Figure 5:**
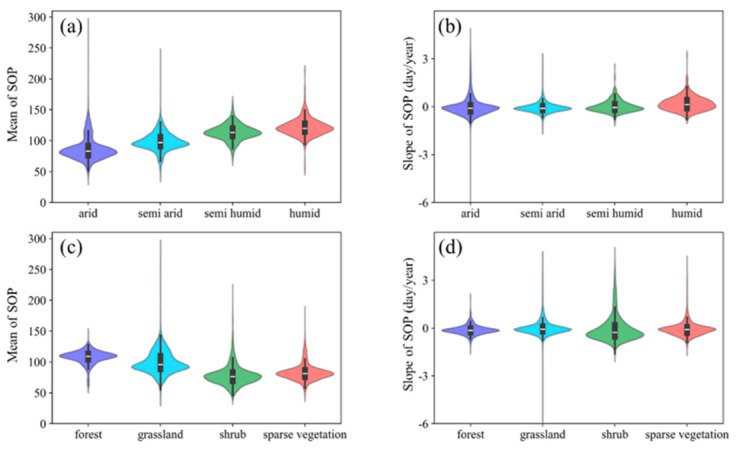
Average SOP distribution of different types of natural vegetation. (**a**) The average SOP distribution of vegetation in all drought index areas, (**b**) the average SOP change trend of vegetation in all drought index areas, (**c**) the average SOP distribution in all vegetation types areas, and (**d**) the average SOP change trend in all vegetation types areas.

The shrubs in the study area are primarily distributed in southern Kazakhstan and southeastern Turkmenistan, where the phenological start time is relatively early and shows little variation across these regions. Steppe meadows, which are widespread in central and northern Kazakhstan, Kyrgyzstan, Tajikistan, and the Tianshan and Kunlun mountain ranges of Xinjiang, China, do not exhibit a clear latitudinal zonality in the mountainous areas of the Tianshan, Kunlun, and Pamir ranges. Sparse vegetation is predominantly found in central and southern Kazakhstan, with smaller patches in southern Turkmenistan, and displays a clear latitudinal zonality (Figure 5).

**Figure 6:**
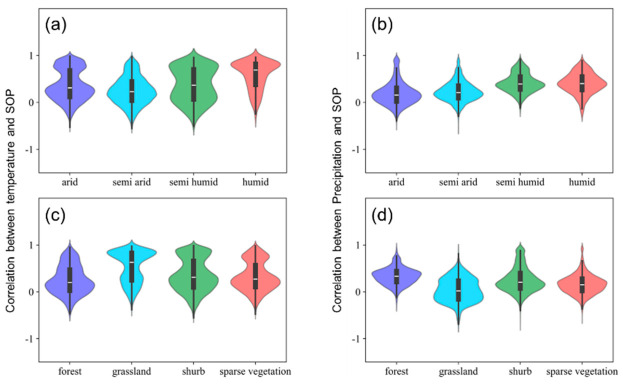
Butterfly map of regional correlation distribution of SOP and meteorological factors in different vegetation types and drought index in the study area (**a**) Correlation between the average SOP of vegetation and temperature in all drought index regions. (**b**) Correlation between the average SOP of vegetation and precipitation in all drought index regions. (**c**) The correlation between the average SOP and temperature in all vegetation type areas. (**d**) The correlation between the average SOP and precipitation in all vegetation type areas.

Figure 6 shows the butterfly diagram of the regional correlation distribution of vegetation SOP and meteorological factors in different vegetation types and drought indices in the study area. Among them, the correlation between vegetation SOP and winter cold accumulation in semi-arid areas is generally weak, while in humid areas, the correlation between vegetation SOP and winter cold accumulation is relatively strong. The correlation distribution between the SOP of vegetation and the accumulation of cold in winter in arid and semi-humid areas is relatively uniform. A trend that is positively correlated with the pre-season average precipitation distribution and the regional humidity level. In different vegetation type regions, the correlation between vegetation SOP and winter cold accumulation in forest areas is relatively weak, while that in grassland areas is relatively strong. The distribution of the correlation between SOP and winter cold accumulation in shrub and sparse vegetation types is relatively uniform. In the comparison of the correlation between SOP and the pre-season average precipitation distribution, there is a relatively weak negative correlation in some grassland and sparse vegetation areas.

**Figure 7:**
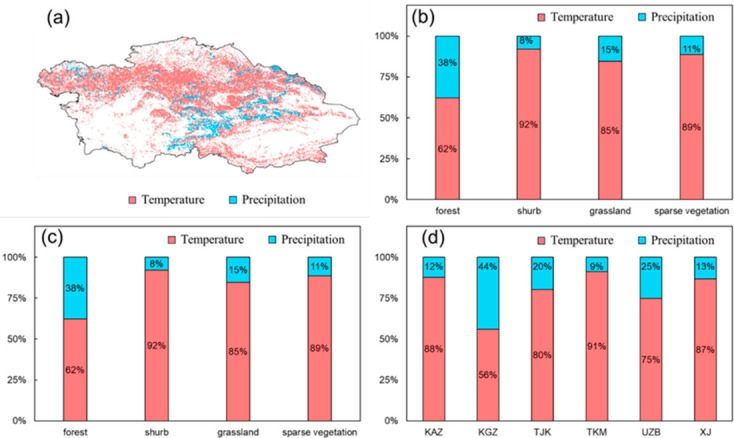
The change in vegetation SOP and the importance of the distribution of meteorological factors in different regions of the study area. (**a**) Influence of temperature and precipitation on the SOP of vegetation (**b**) The proportion of temperature and precipitation as the main influencing factors of vegetation SOP in regions with different drought indices. (**c**) The proportion of temperature and precipitation as the main influencing factors of vegetation SOP in regions with different vegetation types. (**d**) The proportion of temperature and precipitation as the main influencing factors of vegetation SOP in different countries and regions within the study area.

To identify which preseason variable was more closely associated with SOP, the absolute pixelwise correlations of SOP with winter chilling and preseason precipitation were compared. Winter chilling was the dominant correlate across most natural-vegetation pixels, whereas precipitation showed a stronger association in western Kyrgyzstan, western Tajikistan, the Ili Valley of Xinjiang, and the Altai Mountains (Figure 7a). The relative proportions of chilling- and precipitation-dominated pixels were summarized by aridity class, vegetation type, and country or region in Figure 7b–d. Because these results are based on correlations, they represent dominant statistical associations rather than causal importance.

### 3.2. Phenological Trend

In arid Central Asia, the cumulative trend of changes in the start of SOP exhibits a relatively dispersed pattern. Overall, an advancement in SOP is observed in both the eastern and western regions, while a delayed trend predominates in the central areas. For example, the delayed SOP trend is particularly pronounced in the central and southern shrublands of Kazakhstan. In contrast, the western steppe meadows, sparse vegetation regions, and the eastern Altai Mountains show a clear advancement in SOP (Figure 7a). The proportion of pixels exhibiting monotonous changes is relatively low, with 76.8% of the area experiencing shifts in SOP trends over the past 40 years. This indicates a more dispersed spatial distribution of phenological changes compared to the cumulative trend shifts.

Linear regression and EEMD describe different aspects of SOP variation. The linear trend represents the average rate of change over the complete 1982–2022 period, whereas the EEMD residual characterizes the nonlinear long-term trajectory. The cumulative trend expresses the change relative to 1982, and the instantaneous rate indicates the direction and magnitude of change in each year. Consequently, a pixel may exhibit an overall advancing linear trend while also experiencing a temporary period of delay or a reversal in trend direction.

**Figure 8:**
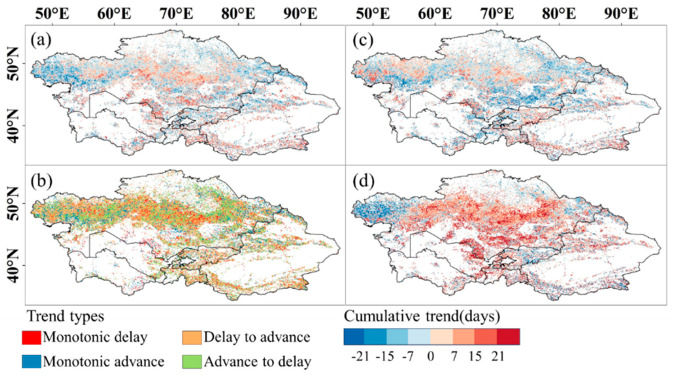
Spatial patterns of cumulative and period-specific SOP change. (**a**) Cumulative SOP change during 1982–2022; (**b**) EEMD-derived trend category; (**c**) SOP trend during 1982–2005; and (**d**) SOP trend during 2006–2022. Negative values indicate advancement, whereas positive values indicate delay.

A widespread transition in the EEMD residual trend was identified around 2005. To characterize this transition, SOP changes were compared between 1982–2005 and 2006–2022 (Figure 8c,d). During 1982–2005, SOP advancement was relatively continuous across the eastern and western parts of the study area. During 2006–2022, the spatial contrast between advancing and delaying trends became more pronounced. Overall, 76.28% of valid pixels experienced at least one change in trend direction, whereas 23.60% maintained a monotonic trend throughout 1982–2022. These findings demonstrate that a single full-period linear slope cannot fully represent the temporal evolution of SOP across much of Central Asia.

The analysis of SOP trends before 2005 demonstrated an improved goodness of fit (R^2^) for forest, shrub, and grassland vegetation types, indicating a more robust alignment of phenological trends with observed data, although the overall improvement for all vegetation types was not statistically significant (Figure 8). Utilizing EEMD, we calculated the nonlinear trends in SOP, revealing a significant shift around 2005. Approximately 76.28% of the pixels showed a change in the SOP trend, while 23.60% of the pixels exhibited a consistent, monotonous SOP trend throughout the period (Table 1).

**Figure 9:**
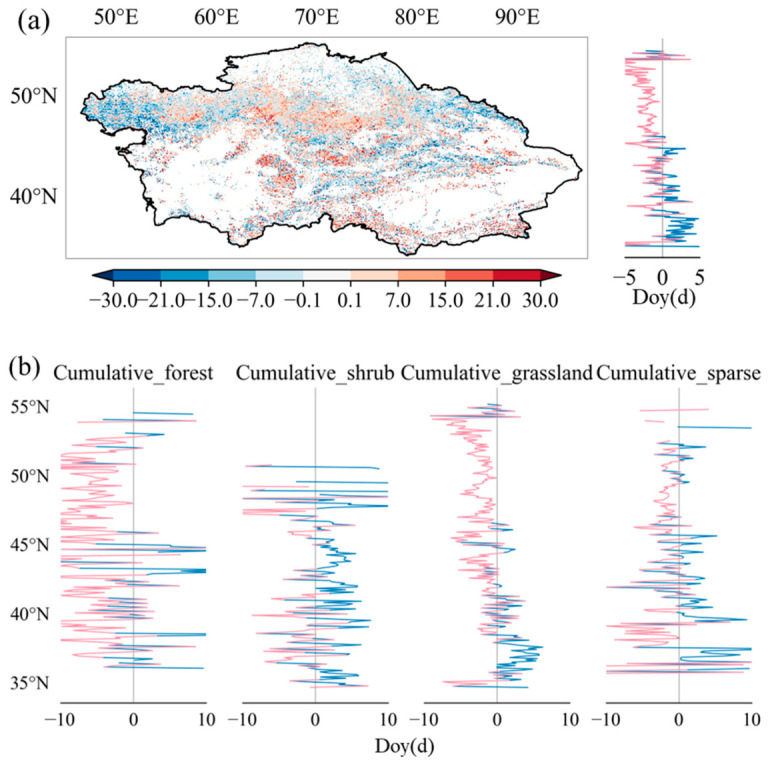
Latitudinal distribution of cumulative changes in the SOP. (**a**) Spatial distribution of cumulative SOP change during 1982–2022 and the corresponding latitudinal profiles for all natural vegetation; (**b**) latitudinal profiles of cumulative SOP change for forest, shrubland, grassland, and sparse vegetation. Within each latitude band, pixels showing advancement and delay were summarized separately. The blue solid lines represent the mean cumulative SOP advancement for pixels with negative cumulative changes, whereas the red lines represent the mean cumulative SOP delay for pixels with positive cumulative changes. The vertical gray line indicates zero change. Negative values indicate earlier SOP relative to 1982, whereas positive values indicate later SOP.

The cumulative SOP change showed a vegetation-dependent latitudinal pattern (Figure 9). To distinguish the coexistence of opposing phenological responses within the same latitude band, pixels with negative and positive cumulative changes were summarized separately. Negative cumulative values represent SOP advancement, whereas positive values represent SOP delay. Advancing pixels were more prevalent and showed greater magnitudes north of 45° N, while delayed SOP was more pronounced south of 39° N. No consistent directional pattern was observed between 39° N and 44° N. The delay in the southern portion of the study area was concentrated mainly in mountain vegetation, whereas sparse vegetation at similar latitudes showed both advancing and delaying responses. These results indicate that latitude alone cannot explain the spatial pattern and that vegetation composition and topography modify the phenological response.

These findings highlight the complexity and variability of phenological responses across different vegetation types and geographic regions in Central Asia. The observed patterns are largely influenced by a range of climatic and environmental factors, emphasizing the diverse ways in which vegetation responds to changing conditions in this arid region.

## 4. Discussion

### 4.1. Overall SOP Advancement and Vegetation-Type Divergence

The average start of photosynthetic activity (SOP) across natural vegetation in Central Asia advanced by 1.26 days per decade during 1982–2022, indicating a general shift toward earlier spring vegetation activity. This finding is consistent with previous regional studies reporting widespread advancement of spring phenology in arid Central Asia under increasing preseason temperatures [33,34]. Wu et al. [33] found that climatic warming altered both vegetation phenology and productivity across arid Central Asia, although the magnitude and direction of the response varied among vegetation and climatic zones. Similarly, Gao and Zhao [34] demonstrated that vegetation phenology in the Great Lakes region of Central Asia was jointly affected by temperature, precipitation, solar radiation, and drought. The overall advancement identified in the present study therefore agrees with the broader regional response to warming but also confirms that a single regional trend conceals considerable vegetation-specific and spatial heterogeneity.

Among the four natural-vegetation classes, forests showed the strongest advancement, at 3.05 days per decade, whereas grassland SOP advanced more moderately, at 1.44 days per decade. One possible explanation for the stronger forest response is that forests in the study area are concentrated mainly in relatively cool northern and montane environments, where spring development may remain strongly limited by thermal forcing. Once winter dormancy requirements have been sufficiently fulfilled, increasing spring temperature can accelerate heat accumulation and produce a pronounced advancement in canopy photosynthetic activity [35,36,37]. Woody vegetation may also maintain a clearer and more stable seasonal NDVI signal than low-cover vegetation, allowing changes in canopy activity to be detected more consistently. Nevertheless, the forest class accounts for a relatively small proportion of the study area, and its distribution is closely associated with elevation, latitude, and regional moisture conditions. The stronger forest trend should therefore not be attributed exclusively to vegetation type without controlling for these spatial covariates.

In contrast, shrubland and sparse vegetation showed slight SOP delays of 0.26 and 0.35 days per decade, respectively. These responses indicate that spring warming does not necessarily produce uniform phenological advancement in water-limited ecosystems. Shrubland and sparse vegetation occur mainly in arid and semi-arid environments where increasing temperature may enhance evaporative demand and intensify soil-water limitation. Under these conditions, the positive influence of thermal accumulation may be partly offset by inadequate preseason moisture. Regional studies have similarly shown that precipitation and drought can be as important as temperature in regulating dryland spring phenology [33,34]. The delayed trends may also reflect reductions in winter chilling, because insufficient chilling can increase the subsequent heat requirement for dormancy release and weaken or reverse the response to spring warming [35,36,37]. Therefore, the divergence among forest, grassland, shrubland, and sparse vegetation likely represents a shift from predominantly temperature-limited phenology in cooler ecosystems toward increasingly moisture- and chilling-constrained phenology in the driest environments.

### 4.2. Interacting Effects of Winter Chilling, Spring Thermal Forcing, and Precipitation

The associations between SOP and preseason climate emphasize that winter chilling and spring thermal forcing should be treated as consecutive but physiologically distinct processes. Exposure to sufficiently low winter temperatures contributes to dormancy release, whereas subsequent warm conditions provide the thermal forcing required for leaf development and the onset of photosynthetic activity. Spring warming generally accelerates heat accumulation and advances phenology, but winter warming may reduce chilling accumulation and thereby increase the forcing requirement. Consequently, warming during winter and warming during spring can have opposing effects on the timing of vegetation activity [35,36,37]. This interaction provides a plausible explanation for why some vegetation types or regions did not show advancement despite an overall increase in surface temperature.

The temperature threshold near 0 °C identified in this study provides an empirical indicator of the transition from winter dormancy to spring heat accumulation. However, it should not be interpreted as a universal physiological dormancy threshold for all vegetation types. Effective chilling and forcing temperatures vary among species, populations, elevations, and climatic regions, and the same temperature may have different effects depending on the amount of chilling already received [35,36,37]. The approximately 0 °C threshold should therefore be described as the regional value that best characterizes the remotely sensed transition in the present analysis. Its predictive reliability should eventually be assessed separately for forest, grassland, shrubland, and sparse vegetation using ground-based phenological or physiological observations.

Although winter chilling showed the stronger association with SOP across most of the study area, preseason precipitation was more strongly associated with SOP in western Kyrgyzstan, western Tajikistan, the Ili Valley, and the Altai Mountains. This pattern is ecologically reasonable because precipitation and snow accumulation affect both preseason soil-water availability and spring surface-energy conditions. Research in the Great Lakes region of Central Asia has demonstrated that spring precipitation and drought can substantially modify phenological responses to temperature [34]. Mountain studies in northwestern Mongolia have likewise shown that spring temperature and winter precipitation jointly regulate spring onset and that their relative influence changes along elevation gradients [38]. Precipitation may thus become a stronger constraint where water availability is limited or where winter snowfall modifies snowmelt timing and the rate of spring soil warming.

Nevertheless, the comparison in this study is based on statistical correlation. A larger absolute correlation with winter chilling or precipitation does not establish that the corresponding variable is the direct causal driver of SOP. Temperature, precipitation, snowfall, radiation, elevation, and soil moisture are interrelated, particularly in mountainous environments. The present results should therefore be described as identifying the dominant climatic correlate, rather than the dominant causal mechanism. Future analyses could strengthen attribution by applying partial correlation, multivariate regression, structural equation modeling, or process-based phenological models that represent chilling, forcing, and moisture simultaneously.

### 4.3. Latitudinal, Elevational, and Topographic Heterogeneity

The results reveal two geographic patterns that should be distinguished conceptually. First, the climatological mean SOP occurred progressively earlier from northern Kazakhstan toward southern Turkmenistan because lower-latitude environments generally reach favorable spring temperatures earlier. Second, the temporal change in SOP during 1982–2022 was characterized by stronger advancement north of 45° N and more frequent delay south of 39° N. These two patterns are not contradictory. Mean SOP describes the average spatial timing of vegetation activity, whereas the temporal trend describes how that timing changed over the study period. Vegetation in southern areas can therefore have an earlier mean SOP while simultaneously showing little additional advancement or even a delayed long-term trend.

The absence of a consistent latitude-dependent pattern in Kyrgyzstan, Tajikistan, and much of Xinjiang indicates that complex topography modifies the broad north–south climatic gradient. Elevation affects temperature, precipitation phase, snow duration, radiation exposure, slope orientation, and soil-water availability, creating strong local differences in spring conditions. Previous studies have reported that spring phenology and its temperature sensitivity vary systematically with elevation and that warming can alter phenological differences among elevation zones [38,39]. Thus, mountain microclimates can override or weaken simple latitudinal controls, particularly in the Tianshan, Pamir, Altai, and Kunlun ranges.

Vegetation at elevations of 1500–3000 m exhibited the strongest SOP advancement, at 2.27 days per decade, compared with 1.52 days per decade below 1500 m. This may indicate that mid-elevation vegetation remains sufficiently temperature limited to respond strongly to spring warming while experiencing less persistent snow and thermal limitation than vegetation at the highest elevations. Comparable elevation-dependent responses have been observed in other mountain regions, where spring temperature and winter precipitation exert different effects at different elevations [38,39]. The result also suggests that the phenological sensitivity of mountain vegetation is not necessarily linear with elevation; instead, the greatest response may occur within an intermediate elevation range where thermal forcing is changing rapidly but vegetation cover remains sufficiently continuous for robust satellite detection.

In contrast, vegetation above 3000 m showed no statistically significant SOP trend. The absence of a significant change should not be interpreted directly as evidence of climatic stability. At high elevations, persistent snow cover, delayed snowmelt, low temperatures, short growing seasons, sparse vegetation, and complex terrain may constrain or obscure the response to warming. Snow cover can substantially alter NDVI trajectories and introduce either positive or negative bias into satellite-derived spring-onset dates depending on snow duration and the end of the snow season [40]. High-elevation pixels are also more likely to contain mixtures of vegetation, rock, bare soil, and snow, which may weaken the correspondence between pixel-level land-surface phenology and actual plant development [41]. Therefore, both ecological constraints and retrieval uncertainty may contribute to the nonsignificant trend above 3000 m.

### 4.4. Nonlinear Transition Around 2005

A major finding of this study is that 76.28% of valid pixels experienced a change in SOP trend direction, whereas only 23.60% maintained a monotonic trend throughout 1982–2022. This result demonstrates that a single full-period linear slope cannot adequately represent the temporal evolution of vegetation phenology across much of Central Asia. A pixel classified as advancing over the complete period may have experienced a temporary delay, a weakening rate of advancement, or a reversal after a particular year. Consequently, reliance only on linear regression may obscure ecologically meaningful transitions.

Ensemble Empirical Mode Decomposition (EEMD) is valuable in this context because it separates oscillatory components from a residual nonlinear trend without requiring a predetermined functional form. The addition and ensemble averaging of white noise reduce mode mixing and improve the separation of temporal scales [32]. Previous phenological research has shown that EEMD can identify trend shifts and time-varying rates that differ substantially from conventional linear estimates [42]. The widespread transition identified around 2005 therefore supports the reviewer’s concern that the interannual trend should not be interpreted solely through the regional linear rate.

The comparison of the periods 1982–2005 and 2006–2022 further showed that the earlier period was characterized by a more spatially continuous advancement, whereas the later period exhibited stronger spatial contrasts between advancing and delaying areas. This change may reflect an altered balance among spring warming, winter chilling, precipitation, drought, and snow conditions. For example, continued winter warming can reduce chilling accumulation and progressively weaken the advancing effect of spring thermal forcing [35,36,37]. Increasing hydroclimatic variability may also generate divergent responses between moisture-limited lowlands and temperature- or snow-limited mountain ecosystems [33,34]. These mechanisms provide plausible interpretations but do not independently demonstrate why the transition occurred around 2005.

The turning point should therefore be regarded as an empirically identified phenological transition rather than being attributed directly to a single climatic event. Stronger attribution would require applying comparable nonlinear or change-point analyses to winter chilling, spring forcing, precipitation, drought, and snow series and then testing whether their turning points coincide spatially and temporally with those of SOP. The robustness of the 2005 transition should also be evaluated against alternative EEMD noise amplitudes, ensemble sizes, endpoint treatments, and independent change-point methods. This is particularly important because the annual series contains only 41 observations, and decomposition-based trends may be sensitive near the beginning and end of a relatively short record.

### 4.5. Limitations and Future Research

Several limitations should be considered when interpreting the results. First, the approximately 8-km NDVI pixels can contain multiple vegetation types and non-vegetated surfaces. Mixed-pixel composition can alter the retrieved green-up date even when the phenology of individual vegetation components remains unchanged [41]. This problem is likely to be particularly important in fragmented mountain landscapes and at the boundaries between grassland, sparse vegetation, bare land, and cropland. Future analyses could apply stricter vegetation-purity thresholds or compare the results with finer-resolution MODIS, VIIRS, Landsat, or Sentinel observations.

Second, applying a static 2020 land-cover map to the entire 1982–2022 period assumes that the land-cover class of each pixel remained unchanged. Historical changes in grazing intensity, cropland extent, irrigation, shrub encroachment, forest disturbance, and vegetation degradation could modify the NDVI seasonal signal and be interpreted incorrectly as phenological change. Stable-land-cover masks derived from multiple years should therefore be used to separate genuine phenological shifts from changes in land-cover composition. This is especially important because simulations have shown that changes in mixed-pixel composition can alter remotely sensed spring dates independently of actual biological phenology [41].

Third, phenological retrieval is intrinsically more uncertain in shrubland and sparse vegetation because low vegetation cover produces small seasonal NDVI amplitudes and stronger soil-background effects. Some of the observed delay in these classes may therefore reflect a combination of ecological response and retrieval uncertainty. In addition, the estimated SOP depends on the selected curve-smoothing algorithm and threshold. Comparative assessments have demonstrated that asymmetric Gaussian, double-logistic, Fourier, and filtering approaches can generate different phenological dates and trends, particularly when time series contain noise or weak seasonality [43]. Sensitivity across smoothing methods and threshold levels should therefore be quantified rather than assuming that one extraction method is universally optimal.

Fourth, snow contamination remains a potential source of uncertainty at northern and high-elevation pixels. Snow can reduce NDVI and generate an artificial increase when melting occurs, thereby shifting the detected SOP either earlier or later depending on the timing of snow disappearance [40]. The pixel-specific low-NDVI replacement procedure used here reduces anomalously low observations but may not completely separate vegetation green-up from snowmelt. Incorporating an independent snow-cover product would allow snow-affected observations to be removed explicitly and would help determine whether the elevation-dependent trends remain stable.

Fifth, ERA5 provides spatially and temporally complete meteorological fields, but it is a model-based reanalysis constrained by observations rather than a direct measurement at every pixel. Its representation of local temperature and precipitation may be less accurate in complex mountain terrain and data-sparse regions [44]. Resampling ERA5 data to the NDVI grid does not create additional meteorological detail and may smooth local climatic gradients. Comparisons with station observations or higher-resolution regional climate datasets would help quantify this uncertainty.

Finally, extensive ground-based observations were unavailable for direct validation of the satellite-derived SOP and the inferred 0 °C threshold. Large-scale land-surface phenology represents an integrated canopy and landscape signal and does not necessarily correspond exactly to leaf unfolding or flowering dates observed for individual species [41,45]. Future studies should combine satellite observations with phenological cameras, field observations, eddy-covariance measurements, or higher-resolution imagery. Such validation is particularly important for forests, sparse vegetation, and snow-affected mountain ecosystems.

Future projections should integrate winter chilling, spring thermal forcing, precipitation, snow dynamics, and vegetation-specific moisture limitation rather than applying a uniform temperature response. The historical relationships identified in this study provide an empirical foundation for developing such models, but reliable future estimates would require calibration and validation, bias-corrected climate-model inputs, multiple emission scenarios, and explicit quantification of uncertainty arising from climate models, vegetation parameters, phenological extraction, and future land-cover change. Chilling should be represented carefully because models that mischaracterize the relationship between chilling accumulation and spring heat requirements may overestimate future phenological advancement [37].

The present analysis focuses on historical phenological changes and does not project SOP under future climate scenarios. Future projection would require a phenological model that explicitly represents the interacting effects of winter chilling, spring thermal forcing, precipitation, snow conditions, and vegetation type. Such a model could subsequently be driven by bias-corrected climate projections from different emission scenarios to estimate future changes in SOP and their uncertainty. The historical relationships and nonlinear transition patterns identified in this study provide an empirical basis for developing such projections. However, uncertainties arising from climate models, emission scenarios, phenological parameterization, land-cover change, and remote-sensing retrieval should be quantified before future SOP estimates are used for ecological management.

Several limitations should be considered. First, the approximately 0.0833° NDVI grid contains mixed land-cover signals, particularly in fragmented mountain environments. Second, applying a static 2020 land-cover map to the complete 1982–2022 period assumes that vegetation classes remained unchanged. Third, SOP retrieval is less reliable in sparse vegetation because of low seasonal NDVI amplitude and stronger soil-background effects. Fourth, phenological dates depend on the selected smoothing method and threshold definition. Fifth, uncertainties in meteorological reanalysis and spatial resampling propagate into the climate–phenology relationships. Sixth, extensive field observations were unavailable for direct regional validation. Finally, correlation identifies association but does not establish causality.

Future research should use stable-land-cover masks, quantify uncertainty across smoothing and threshold combinations, and validate remotely sensed SOP using field observations, phenological cameras, or higher-resolution satellite products. Process-based models jointly representing winter chilling, spring thermal forcing, snow dynamics, and moisture availability would provide stronger mechanistic understanding. Coupling these models with future climate scenarios would permit probabilistic projections of vegetation phenology.

## 5. Conclusions

Natural vegetation in Central Asia exhibited an overall advancement in the start of photosynthetic activity from 1982 to 2022, but the response was neither spatially uniform nor temporally linear. Forests advanced most strongly, grasslands advanced more moderately, and shrubland and sparse vegetation showed slight delays. Contrasting trends north and south of 45° N, pronounced advancement at elevations of 1500–3000 m, and the absence of a significant trend above 3000 m demonstrate that vegetation type, latitude, elevation, and topography jointly shape regional phenology. EEMD further showed that most pixels changed trend direction, with a widespread transition around 2005.

These findings indicate that phenological prediction in arid Central Asia should represent winter chilling, spring thermal forcing, and moisture limitation jointly rather than relying on a single linear temperature response. Vegetation- and region-specific models, supported by field validation and future climate scenarios, are needed to improve ecological forecasting and climate-adaptation planning.

## Figures and Tables

**Table 1:** Variation trend of SOP of vegetation with different cover types.

	Area (%)			
Trend Type	Monotone Delay	Monotone Advance	First Delay, Then Advance	First Advance and Then Delay
Forest	6.16%	21.88%	46.81%	25.00%
Scrub	11.66%	11.59%	32.28%	44.32%
Steppe	9.53%	14.44%	36.79%	39.09%
Sparse vegetation	9.05%	13.52%	37.78%	39.63%
All	9.51%	14.09%	36.92%	39.36%

## Data Availability

Data are contained within the article.
